# A systematic review of the agreement of recall, home-based records, facility records, BCG scar, and serology for ascertaining vaccination status in low and middle-income countries

**DOI:** 10.12688/gatesopenres.12916.2

**Published:** 2020-02-03

**Authors:** Emily Dansereau, David Brown, Lena Stashko, M. Carolina Danovaro-Holliday

**Affiliations:** 1 Strategic Information Group, Expanded Program on Immunization (EPI), Department of Immunizaztion, Vaccines and Biologicals (IVB), World Health Organization, Geneva, Switzerland; 2 Brown Consulting Group Int'l LLC, Cornelius, NC, USA

**Keywords:** immunization, vaccination, LMIC, coverage, survey, methodology, concordance, agreement, validity

## Abstract

**Background:** Household survey data are frequently used to estimate vaccination coverage - a key indicator for monitoring and guiding immunization programs - in low and middle-income countries. Surveys typically rely on documented evidence from home-based records (HBR) and/or maternal recall to determine a child’s vaccination history, and may also include health facility sources, BCG scars, and/or serological data. However, there is no gold standard source for vaccination history and the accuracy of existing sources has been called into question.

**Methods and Findings:** We conducted a systematic review of literature published January 1, 1975 through December 11, 2017 that compared vaccination status at the child-level from at least two sources of vaccination history. 27 articles met inclusion criteria. The percentage point difference in coverage estimates varied substantially when comparing caregiver recall to HBRs (median: +1, range: -43 to +17), to health facility records (median: +5, range: -29 to +34) and to serology (median: -20, range: -32 to +2). Ranges were also wide comparing HBRs to facility-based records (median: +17, range: -61 to +21) and to serology (median: +2, range: -38 to +36). Across 10 studies comparing recall to HBRs, Kappa values exceeded 0.60 in 45% of comparisons; across 7 studies comparing recall to facility-based records, Kappa never reached 0.60. Agreement varied depending on study setting, coverage level, antigen type, number of doses, and child age.

**Conclusions:** Recall and HBR provide relatively concordant vaccination histories in some settings, but both have poor agreement with facility-based records and serology. Long-term, improving clinical decision making and vaccination coverage estimates will depend on strengthening administrative systems and record keeping practices. Short-term, there must be greater recognition of imperfections across available vaccination history sources and explicit clarity regarding survey goals and the level of precision, potential biases, and associated resources needed to achieve these goals.

## Introduction

Vaccination coverage estimates are frequently used at the sub-national, national, and global levels to track performance, set priorities, make managerial and strategic decisions, and allocate funding for immunization programs ^1^. In some cases, vaccination coverage is continuously monitored through child-level registries, but these administrative sources are often unreliable, particularly in low and middle-income countries (LMIC) ^2^. Therefore, LMICs frequently complement administrative recording and reporting data with vaccination coverage surveys, which typically rely on documented evidence in home-based records (HBR) and/or caregiver recall to ascertain a child’s vaccination history ^3– 5^. In some cases, surveys also consult facility records, check for BCG scars, or analyze serological samples for evidence of immunity or prior vaccination ^6, 7^. However, there is no single gold standard for validating whether a child has been vaccinated and the accuracy of these sources for informing coverage estimates remains uncertain.

Multiple factors can cause each vaccination history source to over- or under-estimate coverage ^8^. Caregivers may over-report recalled vaccination histories due to social desirability bias or be unable to recall which and how many vaccinations their children received, particularly as vaccination schedules become more complex ^9, 10^. HBRs can be inaccurate if the record was not brought to every vaccination appointment or the provider made recording mistakes, including failing to record doses, recording doses that were not administered, or misrecording the vaccination date. Facility-based registries and records can be similarly incomplete. BCG vaccination typically leaves a characteristic scar as an indicator of vaccination; however 17 to 25% of vaccinated children may not develop a scar, independent of whether they develop immunity ^11^. Finally, while some consider serology the gold standard for measuring immunity to a disease, this differs conceptually from measuring receipt of a vaccine ^12, 13^. Immunization and vaccination status can differ for multiple vaccine or host-related factors including natural infection, lack of immune response to a vaccine, waning immunity, or deactivation of vaccines due to exposure to extreme temperatures ^7^. Furthermore, some serological assays may misclassify true immunization status due to innate performance limitations. Nevertheless, serological information can inform vaccination coverage estimates, particularly when it is possible to rule out or distinguish natural infection (tetanus, hepatitis B) or in settings where a disease has been eliminated (measles, rubella, or polio).

A review conducted by Miles *et al*. synthesized the literature comparing vaccination history obtained from HBR and recall to health provider-based sources for 1975–2011 ^14^. Compared to provider records, this review found that HBRs under-estimated coverage by a median of 13 percentage points (PP) (range: 61 PP lower to 1 PP higher), while recall over-estimated coverage by a median of 8 PP (range: 58 PP lower to 45 PP higher). The authors concluded that “household vaccination information may not be reliable, and should be interpreted with care.” A review of five studies reporting on validity of caregiver recall (three of the studies were also included in the review by Miles *et al*. ^14^) conducted by Modi and colleagues observed mixed evidence regarding the its usefulness compared to documented evidence of vaccination history in HBRs ^15^. Most importantly, however, only five of 45 articles in the Miles and associates’ review (and the two unique studies identified by Modi and colleagues) were conducted in LMICs. Given that immunization programmes located in LMICs are often the most reliant on survey data to help monitor programme performance and have the highest burden of vaccine-preventable diseases, the authors urged further research in these settings. Extending the inclusion criteria to include more sources of vaccination history and adding research from recent years provides a larger body of evidence from LMICs that should be analyzed. Furthermore, in a 2017 consultation by the World Health Organization (WHO), better understanding the reliability of recall was defined as one of the high research priorities around immunization ^16^.

We conducted a systematic review on the agreement between recall, HBR, health facility sources, BCG scars, and serological data in LMICs. We also investigated how agreement between these sources varies depending on factors including the type of vaccine, number of doses for a given vaccine, age of the child, and total doses in the country’s vaccination schedule.

## Methods

### Literature search

We searched Medline and EMBASE for articles published from January 1, 1975 (aligned to the start of the EPI) through December 11, 2017. The search was restricted to human-related publications and included all languages. We adapted the search terms from the Miles *et al.* review to include additional terms about serology, and restricted to articles with an immunization/vaccination term in the title. We verified that all articles analyzed in the Miles review were found by our search. Articles needed to contain at least one term from each of the following three categories:

- An immunization term in the title: immunization*, immunisation*, vaccin*;
- An agreement term in the title, abstract, MeSH terms or keywords: accuracy, bias, valid*, reliab*, misclassification, error, overestimate*, underestimate*, concordance, agreement, sensitivity, specificity, predictive value, comparing*, compare*, comparison*, authentic*;
- A vaccination history term in the title, abstract, MeSH terms or keywords: recall, remember, medical record*, provider record*, hospital record*, clinical record*, immunization record*, immunisation record*, administrative, card, cards, health booklet, health passport, maternal, parent*, caregiver, mothers, registry, registries, register*, household record*, vaccination record*, serosurvey, seroprevalence, serosurveillance, serological, biomark*, scar*.

Reviews and meta-analyses were not eligible, but their reference lists were manually reviewed, as were the references of each eligible article. We consulted with vaccination experts, including researchers and partners who attended an April 2017 WHO meeting on vaccination coverage surveys, to identify additional studies and unpublished analyses ^17^. The review protocol was created with feedback from experts.

The lead author screened all titles and abstracts, then reviewed the full text to confirm eligibility. Studies needed to meet several inclusion criteria. First, the review was restricted to LMIC, defined by the country’s World Bank income classification for the respective years in which the published studies were conducted ^18^. Second, studies needed to report on vaccines administered to children under 5 years of age. Third, eligible studies had to report and/or compare vaccination status at the child-level from at least two sources, including: recall, HBR, a facility-based source, serological data (see details below) or BCG scar. One article used records from a prospective study where mothers reported their children’s vaccinations on a weekly basis; those records were considered as health facility records. Serological studies were only included if the researcher could plausibly distinguish between immunity from vaccination and immunity from disease. This included tetanus, hepatitis B, and measles in non-measles endemic areas (as determined by the authors of each article). We excluded non population-based studies, including vaccine efficacy studies or studies among special populations such as pre-term infants.

Two researchers (ED and LS) independently extracted study meta data, measures of agreement, and findings on factors associated with agreement from each eligible study, using a pre-defined extraction template. Any discrepancies were discussed and reconciled between the two reviewers and the senior author.

### Analysis

We extracted the following measures for each pair of vaccination history sources in each eligible paper: percentage points (PP) difference in coverage (point estimates only), concordance, kappa statistic, sensitivity, specificity, positive predictive value (PPV) and negative predictive value (NPV) ( Table 1). When papers did not explicitly report all measures, we attempted to calculate them using information provided in the papers. For example, if the paper reported a 2x2 table, we were able to calculate the desired measures of agreement, even if the author had not reported these in the paper. Sensitivity, specificity, PPV, and NPV require designating one source as the ‘gold standard’ or reference group; we used the same reference group(s) as chosen by the authors of each paper. However, we reiterate that in most settings there is no true gold standard for vaccination status to use as the reference. Therefore, these metrics should be interpreted as measures of agreement between two potentially flawed sources, as opposed to measures of validity compared to a gold standard.

**Table 1a.** 2×2 table comparing two sources of vaccination history, used to calculate measures of agreement.

		Reference source (sometimes called ‘gold standard’)	
		+	-
Comparator source	+	True positive	False positive
	-	False negative	True negative

**Table 1b.** Definitions of measures of agreement.

Measure	Definition	Calculation
**PP difference in** **coverage**	Difference between coverage level estimated by the two sources	$Coverage_{Comparator}-Coverage_{Reference}$
**Concordance**	% of children with the same vaccination status from both sources	$\frac{True\;Negative+True\;Positive}{Total\;Children}$
***Kappa* statistic**	Measure of concordance that corrects for chance agreements. Interpretation: <0.2 = poor; 0.21-0.4 = fair; 0.41-0.6 = moderate; 0.61-0.8 = substantial; 0.81-1.0 = near perfect	$\frac{Observed\;Agreement-Expected\;agreement}{1-Expected\;Agreement}$
**Sensitivity**	% of children vaccinated according to the reference source that are vaccinated according to the comparator source	$\frac{True\;Positive}{True\;Positive+False\;Negative}$
**Specificity**	% of children unvaccinated according to the reference source that are unvaccinated according to the comparator source	$\frac{True\;Negative}{True\;Negative+False\;Positive}$
**Positive** **predicative** **values**	% of children vaccinated according to the comparator source who were vaccinated according to the reference source	$\frac{True\;Positive}{True\;Positive+False\;Positive}$
**Negative** **predictive values**	% of children unvaccinated according to the comparator source who were unvaccinated according to the reference source	$\frac{True\;Negative}{True\;Negative+False\;Negative}$

For articles reporting on multiple countries or sub-regions within a country, we treated each geographic region as a separate study population.

For articles reporting on multiple age groups, we used the group closest to 12–23 months in the main analyses, and subsequently conducted a separate analysis of how agreement varied for different age groups within a given study.

Similarly, for articles reporting on multiple doses of the same antigen, we present the results for the most commonly reported dosages in the main analysis, and subsequently conducted a separate analysis of how agreement varied for different doses of the same antigen within a given study. The most common antigen-doses were: Bacille Calmette-Guerin (BCG), 1 ^st^ dose Measles-Containing Vaccine (MCV1), 1 ^st^ dose Oral Polio Vaccine (OPV1), and 1 ^st^ and 3 ^rd^ dose Diphtheria Tetanus Pertussis (DTP), including any DTP-containing combination vaccine. When reported, we also included summary measures for if the child was Up to Date (UTD) on vaccinations for their age, according to the definition used in the original study (with the limitation that that variation in age groups across studies could act as a confounder in the UTD metric).

Analyses were conducted using StataSE 15 and R version 3.3.1.

## Results

### Search results

**Figure 1.**
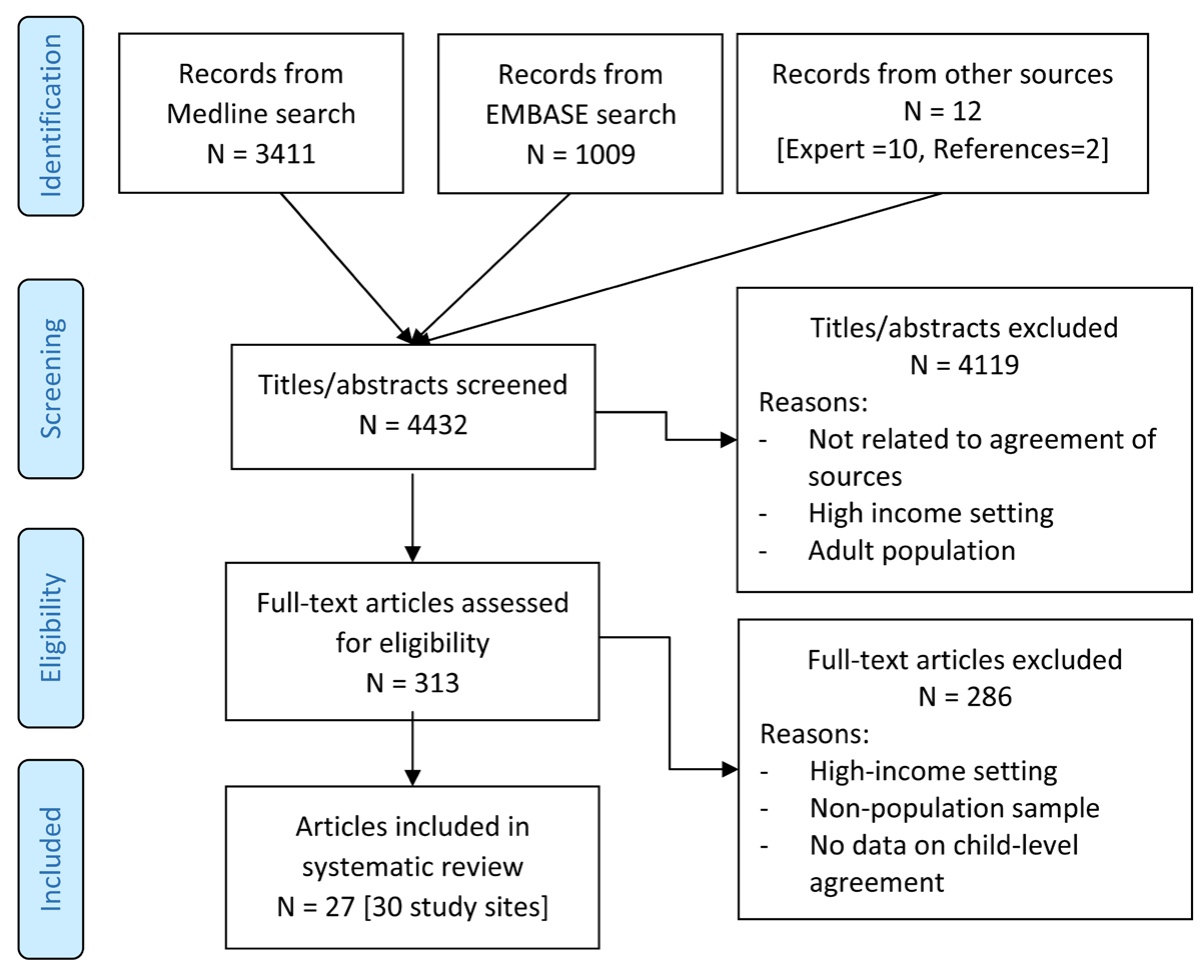
Article screening.

The Medline and EMBASE searches identified a total of 4420 unique titles ( Figure 1). 10 additional titles were identified by experts, and 2 were identified by manually reviewing references. This totaled to 4432 titles, of which 313 passed title and abstract screening and 27 were eligible for the study. Of these, 6 articles were published prior to 2000, 10 from 2000–2009, 8 from 2010–2017, and 3 were unpublished findings provided directly by researchers identified through the expert network ( Table 2). One study contained information on two countries, and one presented results for three sub-national regions, resulting in a total of 30 study sites. 11 study sites were in the World Health Organization (WHO) African region, 5 in the Americas, 4 in the Eastern Mediterranean, 8 in South-East Asia and 2 in Western Pacific ^19^. 15 study sites reported on MCV, 14 on DTP, 10 on BCG, 2 on OPV, and 1 on pneumococcal conjugate vaccine (PCV). Three reported on measures of UTD.

**Table 2.** Articles included in the systematic review.

	First Author	Published	Location	Survey period	Vaccines	Sources of vaccination data
1	Aaby ^20^	1998	Guinea-Bissau	1998	MCV	Facility, recall
2	Adedire ^21^	2016	Nigeria	2013	UTD	HBR, recall
3	Colson ^22^	2015	Mexico, Nicaragua	2012 - 2013	MCV	HBR, serology
4	Dunem ^23^	2010	Angola	2005 - 2006	BCG	HBR, recall, scar
5	GAVI FCE ^24^	Unpublished	Uganda	2015	DTP, PCV	HBR, recall, HBR+recall, serology
6	GAVI FCE ^24^	Unpublished	Zambia	2015	DTP	HBR+recall, serology
7	Gareaballah ^25^	1989	Sudan	1998	MCV	HBR, recall
8	George ^26^	2017	India	2015	DTP	HBR, recall
9	Gong ^27^	Unpublished	Pakistan	2016	MCV	HBR, HBR+recall, serology
10	Hayford ^28^	2013 (author provided data)	Bangladesh	2010 - 2011	BCG, DTP, MCV, OPV	Facility, HBR, recall, HBR+recall, serology
11	Jahn ^29^	2008	Malawi	2002 - 2004	BCG	HBR, scar
12	Langsten ^30^	1998	Egypt	1990 - 1991	BCG, DTP, MCV	HBR, recall
13	Liu ^31^	2017	China	2009 - 2015	MCV	Facility, recall
14	Luman ^32^	2009	N Mariana Islands	2005	UTD	Facility, HBR, recall, HBR+recall
15	Mast ^33^	2006	Uganda	Not given	DTP, MCV	HBR, recall
16	Murhekar ^34^	2017	India	2015	BCG, DTP, MCV, UTD	HBR, recall
17	Nanthavong ^35^	2015	Lao	2013	DTP	HBR, serology
18	Pereira ^36^	2001	Brazil	Not given	BCG	HBR, recall, scar
19	Ramakrishnan ^37^	1999	India	Not given	BCG, DTP, MCV, OPV	Facility, recall
20	Ruiz-Gomez ^38^	2007	Mexico	1999 - 2000	MCV	HBR, serology
21	Selimuzzaman ^39^	2008	Bangladesh	Not given	MCV	HBR, recall
22	Sinno ^40^	2009	Lebanon	2003	UTD	Facility, recall
23	Srisaravanapavananthan ^41^	2008	Sri Lanka	2006	BCG	HBR, scar
24	Tapia ^42^	2006	Mali	Not given	DTP	HBR+facility, serology
25	Travassos ^43^	2016	Ethiopia (3 regions)	2013	DTP	Facility, HBR, recall, serology
26	Ullah ^44^	2000	Bangladesh	Not given	BCG, MCV	Facility, recall
27	Valadez ^45^	1992	Costa Rica	1987	BCG, DTP, MCV, OPV	HBR, recall

### Agreement of sources for all childhood vaccines assessed

**Figure 2.**
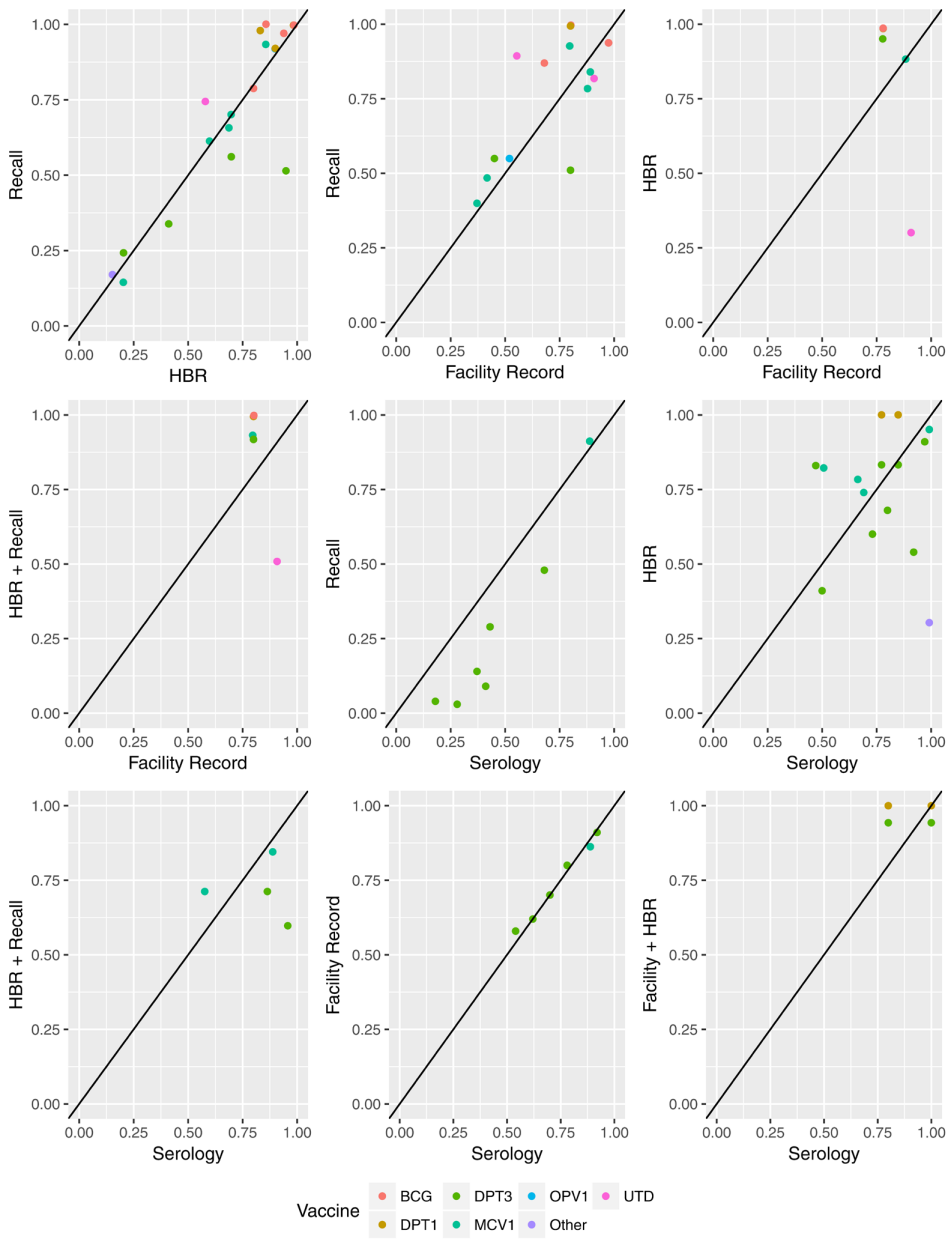
Comparison of vaccination coverage estimates based on different sources of history.

**Figure 3.**
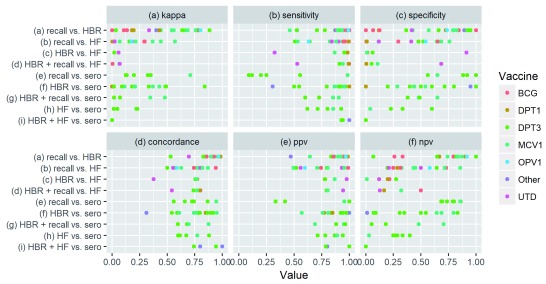
Measures of agreement by source comparison and vaccine.

Recall vs. HBR: Ten papers compared vaccination status based on recall to HBR ( Table 3). The median percentage point difference in coverage estimated using the two was small (1 PP), but ranged from -43 to +17 PP. Recall-based coverage estimates were higher than those based on HBR for 12 of 18 data points, but were only over 10 percentage points higher in 3 cases ( Figure 2). Median kappa (.55) and concordance (.88) between vaccination status based on recall and HBR were substantially higher than any other comparison, and kappa exceeded .60 (“substantial agreement”) 45% of the time ( Figure 3). PPV, sensitivity, NPV and specificity exceeded 80% in 94%, 81%, 56%, and 38% of cases, respectively.

**Table 3.** Summary measures of agreement for standard childhood vaccines and doses, including BCG, DTP3, MCV1, OPV1, PCV1, Yellow Fever (YF) and UTD.

	N articles	N data	PP diff in coverage est.	*Kappa*	Sensitivity	Specificity	Concordance	PPV	NPV
			Median (minimum to maximum)						
**Recall vs. HBR**	10	24	1 (-43 to 17)	0.55 (0.00 to 0.88)	0.95 (0.46 to 1.00)	0.73 (0.00 to 1.00)	0.88 (0.53 to 0.98)	0.93 (0.64 to 0.99)	0.83 (0.07 to 1.00)
**Recall vs. HF**	7	14	5 (-29 to 34)	0.18 (-0.01 to 0.57)	0.89 (0.51 to 1.00)	0.50 (0.00 to 0.76)	0.78 (0.50 to 0.94)	0.80 (0.49 to 0.99)	0.44 (0.20 to 0.86)
**HRB vs. HF**	2	5	17 (-61 to 21)	0.00 (-0.12 to 0.06)	0.95 (0.32 to 0.99)	0.01 (0.01 to 0.91)	0.77 (0.38 to 0.77)	0.78 (0.78 to 0.98)	0.20 (0.01 to 0.27)
**HBR + recall** **vs. HF**	2	5	14 (-40 to 20)	0.01 (-0.05 to 0.07)	0.94 (0.53 to 1.00)	0.05 (0.00 to 0.69)	0.77 (0.54 to 0.80)	0.80 (0.79 to 0.94)	0.17 (0.13 to 0.50)
**Recall vs.** **serology**	2	7	-20 (-32 to 2)	0.26 (0.13 to 0.71)	0.23 (0.09 to 0.99)	0.90 (0.56 to 1.00)	0.73 (0.56 to 0.95)	0.95 (0.33 to 1.00)	0.79 (0.68 to 0.86)
**HBR vs.** **serology**	5	14	2 (-38 to 36)	0.21 (0.00 to 0.84)	0.91 (0.50 to 1.00)	0.44 (0.00 to 1.00)	0.79 (0.54 to 0.95)	0.93 (0.57 to 1.00)	0.52 (0.07 to 0.83)
**HBR + recall vs.** **serology**	3	4	-10 (-36 to 14)	0.21 (0.02 to 0.48)	0.79 (0.60 to 0.91)	0.48 (0.38 to 0.65)	0.69 (0.60 to 0.88)	0.92 (0.69 to 0.96)	0.33 (0.05 to 0.70)
**HF vs. serology**	2	7	0 (-3 to 4)	0.05 (-0.09 to 0.23)	0.80 (0.62 to 0.93)	0.33 (0.04 to 0.60)	0.67 (0.60 to 0.88)	0.87 (0.71 to 0.94)	0.28 (0.03 to 0.40)
**HF + HBR vs.** **serology**	1	4	7 (-6 to 20)	0.00 (-0.1 to 0.00)	0.97 (0.93 to 1.00)	0.00 (0.00 to 0.00)	0.87 (0.74 to 1.00)	0.90 (0.79 to 1.00)	0.00 (0.00 to 0.00)
**HBR vs. scar**	3	3	11 (-4 to 11)	0.08 (0.00 to 0.31)	0.94 (0.85 to 1.00)	0.21 (0.00 to 0.54)	0.89 (0.67 to 0.93)	0.89 (0.74 to 0.98)	0.30 (0.25 to 0.36)
**Recall vs. scar**	1	1	2 (2 to 2)	0.43 (0.43 to 0.43)	0.93 (0.93 to 0.93)	0.48 (0.48 to 0.48)	0.86 (0.86 to 0.86)	0.91 (0.91 to 0.91)	0.54 (0.54 to 0.54)

Recall vs. Facility Records: Seven papers compared recall to health facility records. Recall-based coverage estimates were higher than those based on facility records in 9 of 14 comparisons, 5 of which exceeded +10 percentage points. The median PP difference was +5 PP. Median concordance was .78, and exceeded .80 for 29% of comparisons. Median kappa was .18, and never exceeded .60. Median sensitivity (.85) and PPV (.80) were higher than median specificity (.50) and NPV (.44).

HBR vs. Facility Records: Two papers compared HBR to facility records. Coverage estimates based on HBR were a median of 17 PP higher than those based on facility records, though the range was wide (-61 PP to +21 PP). Most measures of agreement were weak, including a median kappa of 0.00, specificity of 0.01 and NPV of 0.20. Concordance (median=0.77) never exceeded 0.80. Median sensitivity (0.95) and PPV (0.78) were relatively higher.

Recall + HBR vs. Facility Records: The same two studies that compared HBR to facility records also compared combined recall and HBR to facility records, with similar results as those noted above for the HBR vs facility records comparison.

Recall vs. Serology: Two papers including four study sites compared recall to serology. This included one article studying MCV1 vs. measles immunoglobulin G (IgG) and one article (with three study sites) studying pentavalent DTP-Hepatisis B (HepB)- *Haemophilus influenzae* type b (Hib) coverage compared to tetanus IgG and Hib polyribosylribitol phosphate (PRP) antibodies. In the pentavalent DTP-HepB-Hib study, recall consistently under-estimated compared to serology (range: -32 PP to -13 PP), while coverage estimates were similar in the MCV1 study (2 PP higher according to recall). Kappa showed substantial agreement in the measles study (0.71), and ranged from 0.13 to 0.65 in the pentavalent DTP-HepB-Hib study. NPV (median: 0.79, range: 0.68 to 0.86) and specificity (median: 0.90, range: 0.56 to 1.0) were high relative to other types of comparisons, while PPV (0.33 to 1.00) and sensitivity (0.09 to 0.99) varied widely.

HBR vs. Serology: Five papers including eight study sites compared HBR to serology. One study compared DTP to diphtheria and tetanus antibodies, one compared Pentavalent (with DTP as a proxy) to tetanus and Hib antibodies, and three compared to measles antibodies. Coverage based on HBR was a median of 2 PP higher than serologically-confirmed coverage, but the difference ranged from -38 PP to +36 PP. Other measures of agreement also varied widely across the studies and antigens.

Recall + HBR vs. Serology: Three papers compared combined recall and HBR to serology, including two comparing DTP3 to tetanus antibodies and two comparing MCV1 to measles antibodies. Recall + HBR under-estimated DTP3 coverage in both cases (-15 to -36 PP). Recall + HBR over-estimated MCV1 coverage for the one study (+14 PP) and under-estimated in the other (-4 PP). Kappa, sensitivity and NPV were higher in the MCV1 studies than the DTP3 studies.

Facility Records vs. Serology: Two papers containing four study sites compared facility records to serology, including a measles serum study in Bangladesh and a tetanus antibody study in Ethiopia. There was almost no difference in the population-level tetanus estimates for the three sites in Ethiopia (range: -1 to +4 PP) or the measles study in Bangladesh (-3 PP). Kappa was low (median: 0.05, range: -0.09 to 0.23). Sensitivity and PPV tended to be higher than specificity and NPV.

Facility Records + HBR vs. Serology: One paper compared tetanus serum and tetanus oral fluid to combined facility record and HBR information in Mali. In the 12–23 month-old group, it found that the Facility Record + HBR over-estimated coverage compared to the oral tetanus test by 14 PP, but under-estimated by 6 PP compared to the serum. Sensitivity and concordance was high for both, but the kappa and NPV were zero (or nearly zero). 

BCG Scar studies: Four papers reported on BCG scars. Three compared HBR to BCG scars (with scars as the gold standard) and one compared recall to scars. HBR estimated 11 PP higher coverage than scars in one case and 4 PP lower in another, and kappa ranged from 0.00 to 0.31. Sensitivity was high (0.85 to 1.00), but specificity low (0.21 to 0.54). From the one data point available, recall estimated 2 PP higher coverage than scars, with high sensitivity (0.93) but lower specificity (0.48).

### Factors associated with vaccination agreement between data sources

**Figure 4.**
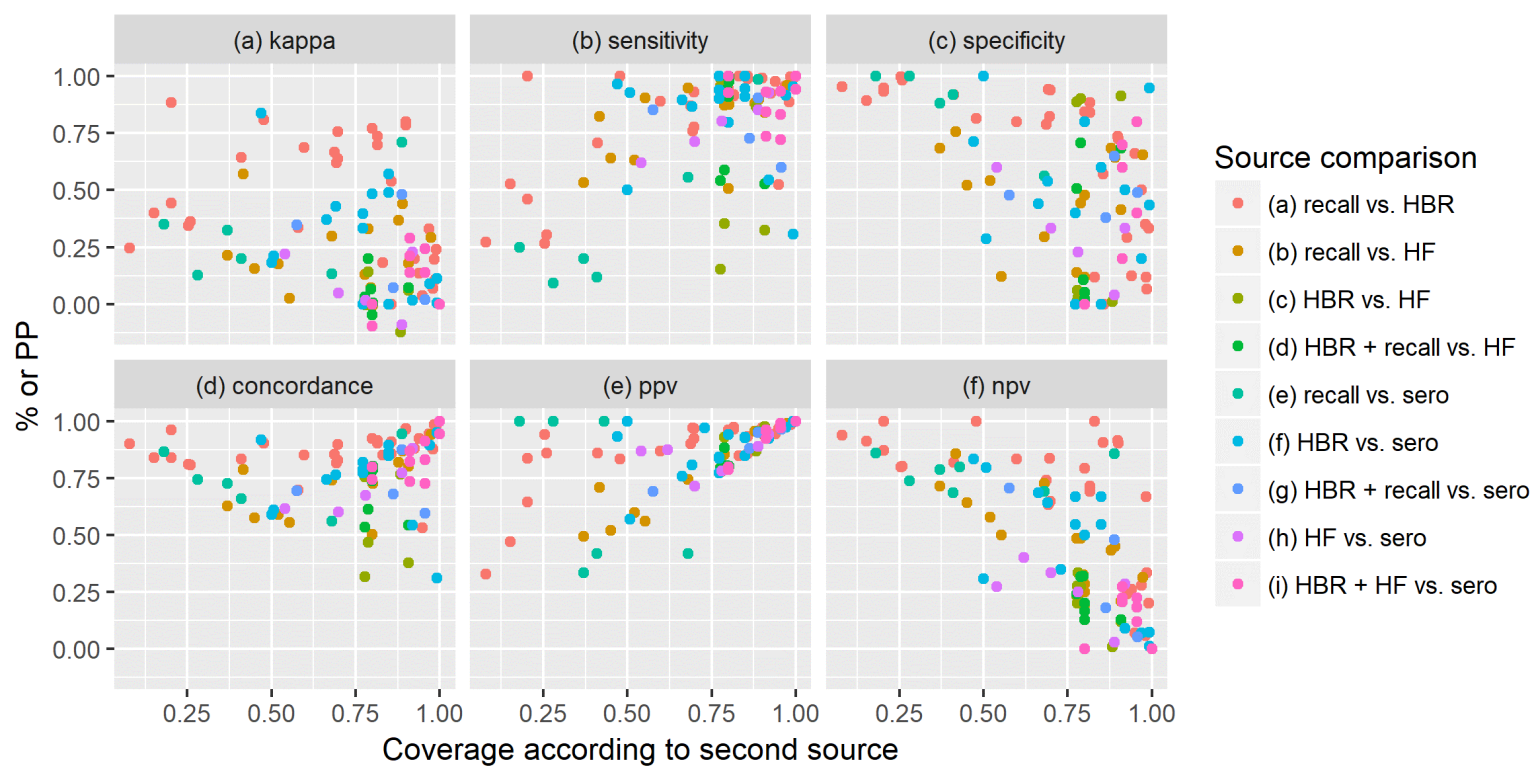
Relationship between coverage level and measures of agreement. HBR: Home-Based Record, HF: Health Facility, PPV: Positive Predictive Value; NPV: Negative Predictive Value; PP: Percentage Point.

Variation by coverage level: When interpreting results, it is important to note that some measures of agreement are inherently affected by the level of vaccination coverage estimated by the reference source. According to mathematical principles, concordance tends to be lowest at 50% coverage and highest at the extremes; PPV increases with coverage; and NPV decreases with coverage. In contrast, kappa, sensitivity and specificity are not affected by vaccination coverage levels. These principles are visibly reflected when comparing agreement measures across studies and vaccines with different coverage levels ( Figure 4). However, there is also confounding by factors such as the study setting, types of sources being compared, and type of vaccine. For example, in settings with >=75% coverage, very few data points report NPV above 0.5, with the exception of some comparing recall to HBR.

**Figure 5.**
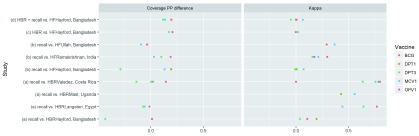
Variation in percentage point difference and kappa for different antigens reported in the same study.

Variation by antigen: Four studies compared recall to HBR for multiple antigens. In all three cases where PP difference could be calculated, DTP3 coverage was underestimated (-45, -14, and –7 PP) more than any other vaccine or dose ( Figure 5). While DTP3 also had the lowest concordance (and BCG the highest), this was explained in part by chance agreement, and no antigen had consistently higher or lower kappa.

Three studies compared recall to facility records for multiple antigens. Two of the studies included DTP3, and DTP3 had the lowest kappa in both (0.50 and 0.57).

**Figure 6.**
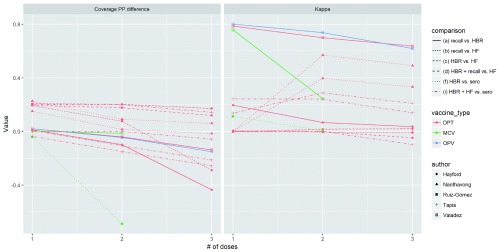
Variation in percentage point difference and kappa for different doses of the same antigen. Each point represents a different number of doses for an antigen, and each line connects points for the same antigen, source and study.

Variation by number of doses: Figure 6 depicts data from five studies that reported on multiple doses of the same antigen, allowing us to analyze how agreement varies by dose. Lines connect points showing a different number of doses for the same antigen, type of comparison, and study site. In nearly all studies, the non-gold standard tends to over-estimate compared to the gold-standard for 1 dose, then come closer to the gold-standard value or even estimate lower coverage than the gold –standard at 2 and 3 doses. Kappa values decrease at higher doses in most studies, with the exception of a study comparing DTP from HBR to diphtheria and tetanus serology in Laos ^35^. Results are level or inconsistent for PPV and NPV across doses.

**Figure 7.**
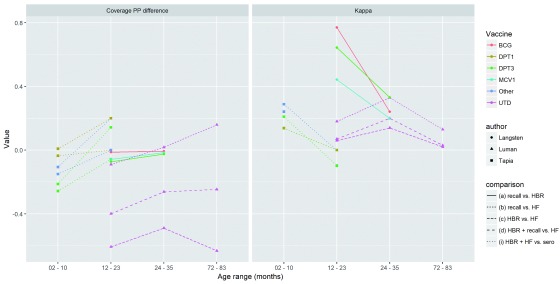
Variation in percentage point difference and kappa by age group. Each point represents an age group for a given antigen/dose. Each line connects points for the same antigen/dose, comparison type and study.

Variation by child age: Figure 7 shows the variation in agreement and recall between sources depending on the age of the child, using data from three of the previously described studies that stratified results for the same vaccine dose by age. Lines connect points showing different age groups for the same vaccine/dose and study site. In the Langsten study, the kappa of recall compared to HBR decreases with age. In the Tapia study, kappa for HBR or health facility record compared to serology decreases with age. In the Luman study, kappa for recall and/or HBR measuring UTD vaccination compared to facility records increase from 12–23 to 24–35 month-olds, but then decrease for 72–83 month-olds.

**Figure 8.**
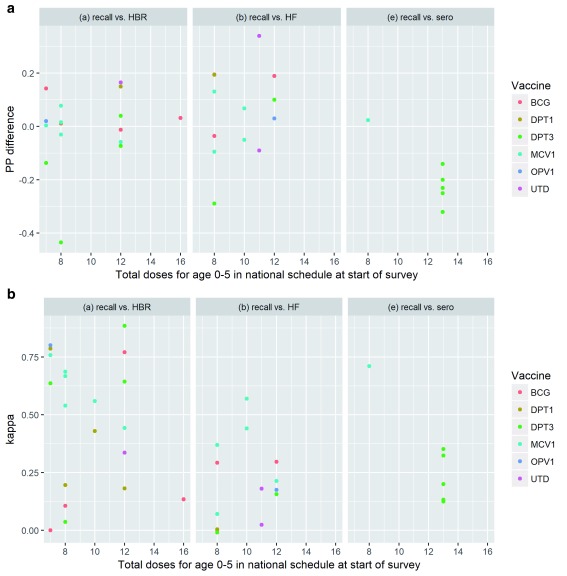
Relationship between number of doses in national schedule and ( **a**) percentage point difference in coverage; ( **b**) kappa.

Variation by schedule complexity: It has been hypothesized that increasingly complex national vaccination schedules reflecting recommendations by WHO ^10^ make it more difficult for caregivers to accurately recall their child’s vaccination history, particularly the number of doses received for multi-dose vaccines. We did not observe a clear, consistent relationship between the number of doses in the national vaccination schedule and the percentage point different in coverage estimates or the kappa statistic for recall as compared to HBR, facility records or serology ( Figure 8) though there were relatively few studies available at periods of time when the national schedule recommended twelve or more vaccines.

Demographic and other factors associated with agreement: Two studies analyzed factors associated with agreement. A study comparing recall to HBR in Costa Rica found that having more doses on the card (correlation coefficient: -0.61) and being an older child (correlation coefficient: -0.35) were associated with smaller error with a p-value<0.0001, while factors including community health worker visits, being recorded in health center records, household size, maternal age and education and socioeconomic status were not significant at the 0.0001 level (specific p-values were not provided) ^45^. In India, a study comparing recall to ongoing prospective reporting found that agreement was higher for younger mothers (1.7 fold increase, p=0.03) ^37^. Other factors including “father's age, sex of the child, place of dwelling, parity, mother's education, family size, previous sibling status and mother's occupation” were not significantly associated with agreement.

## Discussion

Our study finds relatively good agreement between vaccination based on documented evidence in HBRs and that obtained from recall, but comparatively poor agreement versus facility-based records or serology in LMIC settings. Agreement varied substantially depending on the study setting, coverage level, type of antigen, number of doses, and child age.

These findings may be used to heighten awareness and inform discussions about the limitations of survey-based coverage estimates. Survey data have been treated as a ‘gold standard’ to validate or adjust administrative coverage sources, but this assumption may not always be appropriate ^46– 48^. Furthermore, countries with weak administrative systems for coverage estimation are often the same countries where card availability is low and surveys have to rely more on recall ^49^. Those using survey-based vaccination coverage should carefully consider the quality of data underlying the estimates for their specific context(s). For example, current HBR availability has been found to vary considerably across Demographic and Health Surveys (DHS) conducted since 2010 ^50^. Facility registries are also far more complete and accurate in some countries compared to others, and the ease to use them also varies depending on how they are organized (by date of birth, vs date of vaccination visit for example) ^51^. Additionally, while we did not observe that recall validity is changing over time, we believe this remains an open research question, including the influence of different factors including increasing national vaccination schedule complexity ^52^ further complicated by decreasing fertility ^53^ and changing patterns in maternal education ^54, 55^. In order for decision makers to weigh these potential limitations, it is incumbent on those conducting surveys to be clear and thorough in the documentation of their work, including the limitations. Developing a standard template for vaccination coverage survey reports might further support this need for improved transparency.

We also believe additional steps can be taken during the survey design and data collection process to improve available information collected from respondent recall of child vaccination history. For example, DHS and UNICEF Multiple Indicator Cluster Surveys (MICS) currently require respondents to recall the number of doses the child has received for multi-dose vaccines (after obtaining an affirmative response that the child received the multi-dose vaccine). A response of “I don’t know” is most often not available in the standard response set. By requiring a numerical response (e.g., 0, 1, 2, 3 doses), even when the “true” response is “I don’t know”, respondents and enumerators are forced to undertake an ill-understood, unstandardized imputation processes in the field. The classification of “don’t know” responses has been shown to affect coverage estimates by nearly 20 percentage points ^25^. Allowing “don’t know” responses would improve transparency around this important element of uncertainty and empower survey data users to impute in a more systematic way. Surveys might also explore collecting vaccination history from both caregiver recall (asked first of all respondents) and HBRs for all survey respondents, as done in some of the studies included in our review, in order to better assess recall validity among the subset with information from both sources and reveal the directionality and drivers of bias for that particular survey setting.

Despite their limitations and biases, surveys can and will continue to be an important source of information on vaccination programs. As emphasized in the recently updated WHO Survey Reference Manual, surveys will be most useful when they are designed to answer explicit questions ^4^. Clarity about the goals of a survey also gives context to the strengths and limitations of different ascertainment methods and whether additional precision and associated expenses are needed. For example, HBR and recall-based coverage estimates might be considered “good enough” for measuring global or national trends, even if they may over or under-estimate coverage or have poor child-level validity. However, the same data could be inappropriate for measuring achievement against results-based financing goals, as cautioned by the WHO’s Strategic Advisory Group of Experts on Immunization in 2011 ^56^. Greater precision may also be needed to detect change in high-coverage settings ^57^. HBR and recall-based histories could also be problematic if a goal is to monitor equity across socioeconomic groups, as HBR availability and recall bias can vary by the same socioeconomic characteristics that are associated with vaccination coverage; more research is needed on this topic given the recent global emphasis on monitoring equity ^58, 59^. Of course, survey objectives are often more complicated than the examples given here – a survey may have multiple goals or multiple stakeholders each with their own goals. National immunization programs and other survey implementers could benefit from additional WHO guidance about what type of survey design is most appropriate, if at all, given their specific objectives and available data conditions.

Particularly strong clarity about survey goals is needed to justify the added cost and effort of collecting serological samples, as well as to interpreting those findings ^7^. Across included studies, we find substantial discordance between serology and HBR or recall. This is expected given that serology measures something conceptually different than HBR and recall and reinforces that HBR and recall are poor proxies when a survey needs to measure immunization status, as opposed to vaccination status, of a population. Serology has an obvious added value when a decision should be based on population immunity, for example for disease elimination purposes ^13, 60^. However, if the goal is to gather information on vaccination service utilization and dropout, a serosurvey might be difficult and time-consuming to implement and analyze, unnecessary and ultimately wasteful. As methods for collecting and analyzing serology become cheaper, easier and more accurate, researchers and public health officials should continue to explore potential applications, such as using serosurveys to trigger campaigns ^61^.

The intended use of a survey should also guide which specific vaccines are emphasized for analysis and reporting. DTP3 is frequently used as a standard indicator of immunization program performance ^62^. However, DTP3 recall (as compared to HBR and facility sources) is found to have lower concordance and under-estimate coverage by more percentage points than other vaccines in several studies. Therefore, survey users should consider examining other vaccines and doses if precise estimates are needed for decision-making. At the same time, DTP3 may be the most appropriate if the goals are oriented towards measuring delivery and retention in the routine immunization program, given that vaccines such as MCV are often delivered through campaigns in addition to routine immunization. However, the DTP retention metric or dropout (commonly calculated as the relative difference between DTP1 and DTP3 coverage) should still be interpreted with caution given our finding that bias may differ for the 3 ^rd^ versus 1 ^st^ dose.

Finally, the large inconsistencies between home and facility-based records when compared to each other, recall, and serology demonstrate inadequate information for health providers for determining which children have and have not been vaccinated. It is important to be aware that each of these sources is imperfect. Indeed, the primary purpose of these data sources is to serve frontline workers, rather than inform coverage surveys ^63^. Without accurate and complete documentation of children’s vaccination histories, vaccinators will continue to miss opportunities to catch up unvaccinated children as well as waste resources re-vaccinating those who may already be protected ^64^. Such inefficiencies would likely be considered unacceptable in the private sector or other economic fields, and may be overcome using human centered design ^65, 66^ and other innovative approaches to optimize existing immunization programme resources ^67^.

Our study is subject to several limitations. First, although we believe our literature search to be comprehensive, it is possible relevant studies were not identified. In particular, EMBASE and our expert network may not have captured all relevant grey literature. Further, this is an active area of research, and additional studies on the topic have been published since our review cut-off date in December 2017 that provide additional information. As a case in point, a similar yet distinct review of caregiver recall was published as this manuscript was being finalized ^15^. Second, the articles included in our review frequently reported data in inconsistent ways. We made every effort to ensure comparability across studies, but in some cases, we were missing necessary information about methodological or analytical details. For example, not all studies specified how they treated “don’t know” responses from respondents when asked about their child’s vaccination history and there were possible inconsistencies in how different authors counted the dose of polio recommended at birth (polio 0), when in the schedule. We also only focused on point estimates, thus, not taking into account sampling errors. Additionally, we expect there is special difficulty in differentiating vaccination received through routine delivery of vaccination versus campaign doses, including for MCV. As this issue was often not discussed by the source articles, it may not be well-addressed in our study. Most articles also did not document the phrasing of vaccination history recall questions; studying the best way to solicit recall, including the use of visual cues, is an area for future research. Some of these limitations may be addressed through further analysis of existing data, which the researchers approached as part of this review were agreeable to do. Finally, we did not include an assessment of the quality of each study. The level of detail provided about the survey design, data collection, and analysis methods varied substantially across studies. Going forward, the WHO is working to define clearer quality criteria for surveys measuring vaccination coverage, which could serve as a benchmark and standardize reporting. We did take special effort to assess the quality of un-published work before including these in the review, by speaking directly with the researchers to understand the design, implementation, and limitations of their studies. 

In conclusion, while recall and HBR provide relatively concordant vaccination histories in some settings, both have poor agreement when compared to facility-based records and serology. In the long-term, improving clinical decision making for immunization and survey-based vaccination coverage estimates will depend on strengthening administrative systems, recording practices and record keeping. In the short-term, there must be greater recognition of imperfections in current ascertainment techniques, paired with explicit clarity regarding the goals of surveys and the level of precision, potential biases, and associated resources needed to achieve these goals.

## Data availability

### Underlying data

Open Science Framework: A systematic review of the agreement of recall, home-based records, facility records, BCG scar, and serology for ascertaining vaccination status in low and middle-income countries. https://doi.org/10.17605/OSF.IO/S5UBY ^68^

This project contains the following underlying data:

- Supplemental Table 1: List of all articles used in analysis.

### Extended data

Open Science Framework: A systematic review of the agreement of recall, home-based records, facility records, BCG scar, and serology for ascertaining vaccination status in low and middle-income countries. https://doi.org/10.17605/OSF.IO/S5UBY ^68^

This project contains the following extended data:

- Search term syntax

### Reporting guidelines

PRISMA checklist: https://doi.org/10.17605/OSF.IO/S5UBY ^68^

Data are available under the terms of the Creative Commons Zero "No rights reserved" data waiver (CC0 1.0 Public domain dedication).

## References

[1] Duclos P Okwo-Bele JM Gacic-Dobo M: Global immunization: status, progress, challenges and future. *BMC Int Health Hum Rights.* 2009;9(Suppl 1):S2. 10.1186/1472-698X-9-S1-S2 19828060 PMC2762311

[2] Cutts FT Claquin P Danovaro-Holliday MC: Monitoring vaccination coverage: Defining the role of surveys. *Vaccine.* 2016;34(35):4103–9. 10.1016/j.vaccine.2016.06.053 27349841 PMC4967442

[3] UNICEF: MICS6 Questionnaires and Modules.2016 Reference Source

[4] WHO: 2015 Working Draft - New WHO Vaccination Coverage Cluster Survey Manual.2015 Reference Source

[5] Demographic and Health Surveys (DHS): DHS Model Questionnaire - Phase 7.2015 Reference Source

[6] UNICEF: Multiple Indicator Cluster Surveys (MICS) 6: Questionnaire Form for Vaccination Records at Health Facility.2016 Reference Source

[7] Cutts FT Hanson M: Seroepidemiology: an underused tool for designing and monitoring vaccination programmes in low- and middle-income countries. *Trop Med Int Health.* 2016;21(9):1086–98. 10.1111/tmi.12737 27300255

[8] Cutts FT Izurieta HS Rhoda DA: Measuring coverage in MNCH: design, implementation, and interpretation challenges associated with tracking vaccination coverage using household surveys. *PLoS Med.* 2013;10(5):e1001404. 10.1371/journal.pmed.1001404 23667334 PMC3646208

[9] Althubaiti A: Information bias in health research: definition, pitfalls, and adjustment methods. *J Multidiscip Healthc.* 2016;9:211–7. [cited 2017 Oct 10]. 10.2147/JMDH.S104807 27217764 PMC4862344

[10] WHO: WHO recommendations for routine immunization - summary tables. Reference Source

[11] Barreto ML Pereira SM Ferreira AA: BCG vaccine: efficacy and indications for vaccination and revaccination. *J Pediatr (Rio J).* 2006;82(3 Suppl):S45–54. 10.2223/JPED.1499 16826312

[12] MacNeil JR Bennett N Farley MM: Epidemiology of infant meningococcal disease in the United States, 2006-2012. *Pediatrics.* 2015;135(2):e305–311. 10.1542/peds.2014-2035 25583921 PMC4803024

[13] Durrheim DN Orenstein WA Schluter WW: Assessing population immunity for measles elimination - The promise and peril of serosurveys. *Vaccine.* 2018;36(28):4001–3. 10.1016/j.vaccine.2018.04.036 29793892 PMC10299846

[14] Miles M Ryman TK Dietz V: Validity of vaccination cards and parental recall to estimate vaccination coverage: a systematic review of the literature. *Vaccine.* 2013;31(12):1560–8. 10.1016/j.vaccine.2012.10.089 23196207

[15] Modi RN King C Bar-Zeev N: Caregiver recall in childhood vaccination surveys: Systematic review of recall quality and use in low- and middle-income settings. *Vaccine.* 2018;36(29):4161–70. 10.1016/j.vaccine.2018.05.089 29885771

[16] Danovaro-Holliday MC Dansereau E Rhoda DA: Collecting and using reliable vaccination coverage survey estimates: Summary and recommendations from the "Meeting to share lessons learnt from the roll-out of the updated WHO Vaccination Coverage Cluster Survey Reference Manual and to set an operational research agenda around vaccination coverage surveys", Geneva, 18-21 April 2017. *Vaccine.* 2018;36(34):5150–9. 10.1016/j.vaccine.2018.07.019 30041880 PMC6099121

[17] WHO: Meeting Report: Meeting to share lessons learnt from the roll-out of the 2015 WHO Vaccination Coverage Cluster Survey Reference Manual and to set an operational research agenda around vaccination coverage surveys. Reference Source 10.1016/j.vaccine.2018.07.019 PMC6099121 30041880

[18] World Bank: World Bank Country and Lending Groups: Historical Classifications, 2018 FY.2017 Reference Source

[19] WHO: Definition of regional groupings. WHO. [cited 2017 Oct 9]. Reference Source

[20] Aaby P Martins C Balé C: Assessing measles vaccination coverage by maternal recall in Guinea-Bissau. *Lancet.* 1998;352(9135):1229. 10.1016/S0140-6736(05)60575-2 9777875

[21] Adedire EB Ajayi I Fawole OI: Immunisation coverage and its determinants among children aged 12-23 months in Atakumosa-west district, Osun State Nigeria: a cross-sectional study. *BMC Public Health.* 2016;16(1):905. 10.1186/s12889-016-3531-x 27578303 PMC5006522

[22] Colson KE Gagnier MC Palmisano E: Comparative estimates of immunisation coverage from three different sources: results from the SM2015 evaluation. *Lancet.* 2013;381(Supplement 2):S32 10.1016/S0140-6736(13)61286-6

[23] Van-Dunem JC de Alencar LC Rodrigues LC: Sensitivity and specificity of BCG scar reading among HIV-infected children. *Vaccine.* 2010;28(9):2067–9. 10.1016/j.vaccine.2009.12.034 20060085

[24] Gavi Full Country Evaluation Team D: Gavi Full Country Evaluation Data - Uganda and Zambia. Unpublished, provided by author.2017 Reference Source

[25] Gareaballah ET Loevinsohn BP: The accuracy of mother's reports about their children's vaccination status. *Bull World Health Organ.* 1989;67(6):669–74. 2633882 PMC2491322

[26] George K Victor S Abel R: Reliability of mother as an informant with regard to immunisation. *Indian J Pediatr.* 1990;57(4):588–90. 10.1007/BF02726779 2286415

[27] Gong W Hayford K Shah M: Overcoming information biases in vaccination coverage surveys using serum immune marker assessments: A combination of household survey, field blood draw, and modeling analysis. Unpublished, provided by author.2017.

[28] Hayford KT Shomik MS Al-Emran HM: Measles vaccination coverage estimates from surveys, clinic records, and immune markers in oral fluid and blood: a population-based cross-sectional study. *BMC Public Health.* 2013;13:1211. 10.1186/1471-2458-13-1211 24359402 PMC3890518

[29] Jahn A Floyd S Mwinuka V: Ascertainment of childhood vaccination histories in northern Malawi. *Trop Med Int Health.* 2008;13(1):129–38. 10.1111/j.1365-3156.2007.01982.x 18291011

[30] Langsten R Hill K: The accuracy of mothers’ reports of child vaccination: evidence from rural Egypt. *Soc Sci Med.* 1998;46(9):1205–12. 10.1016/S0277-9536(97)10049-1 9572610

[31] Liu G Liao Z Xu X: Accuracy of parent-reported measles-containing vaccination status of children with measles. *Public Health.* 2017;144:92–5. 10.1016/j.puhe.2016.12.013 28274390

[32] Luman ET Ryman TK Sablan M: Estimating vaccination coverage: validity of household-retained vaccination cards and parental recall. *Vaccine.* 2009;27(19):2534–9. 10.1016/j.vaccine.2008.10.002 18948158

[33] Mast TC Kigozi G Wabwire-Mangen F: Immunisation coverage among children born to HIV-infected women in Rakai district, Uganda: Effect of voluntary testing and counselling (VCT). *AIDS Care.* 2006;18(7):755–63. 10.1080/09540120500521053 16971285

[34] Murhekar MV Kamaraj P Kanagasabai K: Coverage of childhood vaccination among children aged 12-23 months, Tamil Nadu, 2015, India. *Indian J Med Res.* 2017;145(3):377–86. 28749402 10.4103/ijmr.IJMR_1666_15 PMC5555068

[35] Nanthavong N Black AP Nouanthong P: Diphtheria in Lao PDR: Insufficient Coverage or Ineffective Vaccine? *PLoS One.* 2015;10(4):e0121749. 10.1371/journal.pone.0121749 25909365 PMC4409043

[36] Pereira SM Dourado I Barreto ML: Sensitivity and specificity of BCG scar reading in Brazil. *Int J Tuberc Lung Dis Off J Int Union Tuberc Lung Dis.* 2001;5(11):1067–70. 11716343

[37] Ramakrishnan R Rao TV Sundaramoorthy L: Magnitude of recall bias in the estimation of immunization coverage and its determinants. *Indian Pediatr.* 1999;36(9):881–5. 10744865

[38] Ruiz-Gómez J Valdespino JL Olaiz-Fernández G: Encuesta serológica nacional del sarampión en niños: evidencias para su eliminación. *Salud Pública México.* 2007;49(S3):370–6. 10.1590/S0036-36342007000900008

[39] Selimuzzaman ABM Ullah MA Haque MJ: Accuracy of Mothers’ Reports Regarding Vaccination Status of Their Children in Urban Bangladesh. *TAJ J Teach Assoc.* 2008;21(1):40–3. 10.3329/taj.v21i1.3217

[40] Sinno DD Shoaib HA Musharrafieh UM: Prevalence and predictors of immunization in a health insurance plan in a developing country. *Pediatr Int.* 2009;51(4):520–5. 10.1111/j.1442-200X.2008.02769.x 19400813

[41] Srisaravanapavanantha N Dissanayake NN Sarathchandra J: BCG vaccination scars of childen under five years in a tertiary care hospital. *Sri Lanka J Child Health.* 2008;37(3): [cited 2018 Sep 16]. 10.4038/sljch.v37i3.106

[42] Tapia MD Pasetti MF Cuberos L: Measurement of tetanus antitoxin in oral fluid: a tool to conduct serosurveys. *Pediatr Infect Dis J.* 2006;25(9):819–25. 10.1097/01.inf.0000232629.72160.bb 16940841

[43] Travassos MA Beyene B Adam Z: Immunization Coverage Surveys and Linked Biomarker Serosurveys in Three Regions in Ethiopia. *PLoS One.* 2016;11(3):e0149970. 10.1371/journal.pone.0149970 26934372 PMC4774907

[44] Ullah MA Barman A: Validity of mothers’ statements about their children’s vaccination status in rural Bangladesh. *Int Med J.* 2000;7:93–6. Reference Source

[45] Valadez JJ Weld LH: Maternal recall error of child vaccination status in a developing nation. *Am J Public Health.* 1992;82(1):120–2. 10.2105/AJPH.82.1.120 1536315 PMC1694427

[46] Murray CJ Shengelia B Gupta N: Validity of reported vaccination coverage in 45 countries. *Lancet.* 2003;362(9389):1022–7. 10.1016/S0140-6736(03)14411-X 14522532

[47] Burton A Kowalski R Gacic-Dobo M: A formal representation of the WHO and UNICEF estimates of national immunization coverage: a computational logic approach. *PLoS One.* 2012;7(10):e47806. 10.1371/journal.pone.0047806 23133527 PMC3485034

[48] Lim SS Stein DB Charrow A: Tracking progress towards universal childhood immunisation and the impact of global initiatives: a systematic analysis of three-dose diphtheria, tetanus, and pertussis immunisation coverage. *Lancet.* 2008;372(9655):2031–46. 10.1016/S0140-6736(08)61869-3 19070738

[49] Cutts FT Claquin P Danovaro-Holliday MC: Reply to comments on Monitoring vaccination coverage: Defining the role of surveys. *Vaccine.* 2016;34(50):6112–3. 10.1016/j.vaccine.2016.09.067 27899197 PMC5142421

[50] Brown DW Gacic-Dobo M: Home-based record prevalence among children aged 12-23 months from 180 demographic and health surveys. *Vaccine.* 2015;33(22):2584–93. 10.1016/j.vaccine.2015.03.101 25887089

[51] Bosch-Capblanch X Ronveaux O Doyle V: Accuracy and quality of immunization information systems in forty-one low income countries. *Trop Med Int Health.* 2009;14(1):2–10. 10.1111/j.1365-3156.2008.02181.x 19152556

[52] Loharikar A Dumolard L Chu S: Status of New Vaccine Introduction - Worldwide, September 2016. *MMWR Morb Mortal Wkly Rep.* 2016;65(41):1136–1140. 10.15585/mmwr.mm6541a3 27764083

[53] United Nations, Department of Economic and Social Affairs, Population Division: World fertility patterns 2015 - Data booklet.2015 Reference Source

[54] Gakidou E Cowling K Lozano R: Increased educational attainment and its effect on child mortality in 175 countries between 1970 and 2009: a systematic analysis. *Lancet.* 2010;376(9745):959–74. 10.1016/S0140-6736(10)61257-3 20851260

[55] Ngandu NK Manda S Besada D: Does adjusting for recall in trend analysis affect coverage estimates for maternal and child health indicators? An analysis of DHS and MICS survey data. *Glob Health Action.* 2016;9:32408. 10.3402/gha.v9.32408 27829489 PMC5102105

[56] Strategic Advisory Group of Experts on Immunization (SAGE): Meeting of the Strategic Advisory Group of Experts on Immunization, November 2011 - Conclusions and Recommendations. Reference Source

[57] Brown DW Burton AH Feeney G: Avoiding the Will O’ the Wisp: Challenges in Measuring High Levels of Immunization Coverage with Precision. *World J Vaccines.* 2014;4(3):97–99. 10.4236/wjv.2014.43012

[58] GAVI: 2016-2020 Strategy Indicator Definitions.2016 Reference Source

[59] WHO: State of inequality: Childhood immunization. WHO. [cited 2018 Sep 16]. Reference Source

[60] World Health Organization. Electronic address: sageexecsec@who.int: Tetanus vaccines: WHO position paper, February 2017 - Recommendations. *Vaccine.* 2018;36(25):3573–3575. 10.1016/j.vaccine.2017.02.034 28427847

[61] Lessler J Metcalf CJ Cutts FT: Impact on Epidemic Measles of Vaccination Campaigns Triggered by Disease Outbreaks or Serosurveys: A Modeling Study. *PLoS Med.* 2016;13(10):e1002144. 10.1371/journal.pmed.1002144 27727285 PMC5058560

[62] Global Vaccine Action Plan 2011-2020. WHO.2013[cited 2017 Oct 11]. Reference Source

[63] Hasman A Rapp A Brown DW: Revitalizing the home-based record: Reflections from an innovative south-south exchange for optimizing the quality, availability and use of home-based records in immunization systems. *Vaccine.* 2016;34(47):5697–5699. 10.1016/j.vaccine.2016.09.064 27743647

[64] Hanson CM Mirza I Kumapley R: Enhancing immunization during second year of life by reducing missed opportunities for vaccinations in 46 countries. *Vaccine.* 2018;36(23):3260–3268. 10.1016/j.vaccine.2018.04.070 29731113

[65] Bazzano AN Martin J Hicks E: Human-centred design in global health: A scoping review of applications and contexts. *PLoS One.* 2017;12(11):e0186744. 10.1371/journal.pone.0186744 29091935 PMC5665524

[66] PHISICC. [cited 2017 Dec 23]. Reference Source

[67] Shifo Foundation: MyChild Card Evaluation Report.2016 Reference Source

[68] Dansereau E: A Systematic Review of the Agreement of Recall, Home-Based Records, Facility Records, BCG Scar, and Serology for Ascertaining Vaccination Status in Low and Middle-Income Countries.OSF.2019 10.17605/OSF.IO/S5UBY PMC7110941 32270134

