# Supplementary material for: A systematic review of the agreement of recall, home-based records, facility records, BCG scar, and serology for ascertaining vaccination status in low and middle-income countries
Source: Gates Open Res. 2020 Feb 3;3:923. Originally published 2019 Mar 21. [Version 2] doi: 10.12688/gatesopenres.12916.2 (PMC7110941; doi:10.12688/gatesopenres.12916.2)
Supplement: Peer review history

# Peer review: Reviewer response for version 2

**DOI:** 10.21956/gatesopenres.14264.r28524

**Published:** 2020-03-30

**Authors:** Charles Shey Wiysonge

**Affiliations:** 1 Cochrane South Africa, South African Medical Research Council, Cape Town, South Africa

I read the revised version of the systematic review by Emily Dansereau and colleagues with great interest. The authors conducted a systematic review of peer-reviewed literature published from 01 January 1975 to 11 December 2017. They found that vaccination data from caregiver recall were concordant with those from home-based records; but both had poor agreement with coverage data from facility-based records and serology. They took all our previous comments into consideration when revising the article and I have no further comments. This is a policy- and research-relevant publication. Well done.

I confirm that I have read this submission and believe that I have an appropriate level of expertise to confirm that it is of an acceptable scientific standard.

# Peer review: Reviewer response for version 1

**DOI:** 10.21956/gatesopenres.14015.r27015

**Published:** 2019-04-24

**Authors:** Charles Shey Wiysonge, Olatunji Adetokunboh

**Affiliations:** 1 Cochrane South Africa, South African Medical Research Council, Cape Town, South Africa; 2 Department of Global Health, Faculty of Medicine and Health Sciences, Stellenbosch University, Cape Town, South Africa

We commend Emily Dansereau and colleagues for conducting this timely systematic review on the agreement between recall, home-based records, health facility sources, BCG scars, and serological data in low and middle-income countries. While we applaud the efforts of the authors, we think the review could be improved, as follows:

1. The authors indicate that they prepared a protocol for the review but have not shared it. It is important to provide a link to the protocol. Was the protocol for the review registered in PROSPERO or similar platform? What sort of studies were expected to be included in the review? Was there any deviation from the protocol at the end of the research?
2. What informed the use of 1 ^st^ January 1957 as the cut-off date for the search?
3. Given the increased research activity on immunization data, a search conducted up to December 2017 should be considered out of date for a paper submitted in March 2019. If the search is not updated, the authors should clearly identify this as a limitation.
4. Quality assessment of included studies is essential. We cannot make a sound conclusion without knowing the quality of the included studies. If this is not done, the authors should identify this as a limitation.
5. Are there particular reasons for not searching for grey literature? 
6. In studies with multiple antigens, what informed the choice of antigen that was included in the analysis?
7. The authors should provide the complete search strategy used for one of the two databases, preferably Medline.

We confirm that we have read this submission and believe that we have an appropriate level of expertise to confirm that it is of an acceptable scientific standard, however we have significant reservations, as outlined above.

# Response

**Published:** 2019-12-21

**Authors:** Emily Dansereau

We thank the reviewers very much for the time they have given to review the article and provide this constructive feedback.

*1. The authors indicate that they prepared a protocol for the review but have not shared it. It is important to provide a link to the protocol. Was the protocol for the review registered in PROSPERO or similar platform? What sort of studies were expected to be included in the review? Was there any deviation from the protocol at the end of the research?*

 Thank you for noting the omission of our protocol. We have now added this to the OSF storage platform. We did not register it in a formal protocol platform; that is a good point of feedback for the future. We sought and received input from 20 experts on the protocol, including content matter experts as well as library science professionals. Our review and protocol were designed as an update and extension of the previous publication by Miles et al, which reviewed a similar set of literature through 2011. Therefore we expected that the publications would be of similar nature to those in the Miles study -- however we extended our inclusion/exclusion criteria to capture a broader set of studies, by expanding the search terms, notably including to include data from serological surveys. We were able to stick fairly closely to our original protocol. We note the three deviations. First, we had originally thought to apply a slightly different search strategy for the period already covered by the Miles review (more narrow), and the period after (more broad) - however in the end we decided it was more comprehensive and consistent to apply a single set of broad search terms across the entire time period of interest. Second, though the protocol noted we would look for ‘peer-reviewed published literature’ this statement was inconsistent with the proposed search approach in the same protocol which included searching EMBASE, which includes grey literature including conference abstracts, and drawing on our expert network’s knowledge of unpublished work – the intention was always to include these sources. We opted to include 3 unpublished studies in the end – (two from the Gavi Full Country Evaluations and one from an established research group working in Pakistan). The final change from original protocol was on the analysis side: after conducting the review, we decided it was not fitting to conduct a regression-based meta-analysis of the results as proposed, due to the heterogeneous nature of the studies included. We instead opted to focus on displaying and visualizing the results in a way that maximized interpretability for the readers, and synthesizing them descriptively.

*2. What informed the use of 1st January 1957 as the cut-off date for the search?*

 Thank you for this question, and for catching an important typo in the manuscript. The start date was 1st January 1975, and date this was chosen because it aligned with the establishment of the EPI program. This has been corrected and explained in the updated manuscript.

*3. Given the increased research activity on immunization data, a search conducted up to December 2017 should be considered out of date for a paper submitted in March 2019. If the search is not updated, the authors should clearly identify this as a limitation.*

 We agree and are glad to see this is an active area of research. This has now been noted as a limitation.

*4. Quality assessment of included studies is essential. We cannot make a sound conclusion without knowing the quality of the included studies. If this is not done, the authors should identify this as a limitation.*

 Thank you for raising this important point. While we would have preferred to assess the study quality, it was difficult to do so with the information provided. WHO has work going forward to more clearly define quality criteria for coverage surveys, which would also help standardize the reporting. For the unpublished studies, we did take special effort to assess the quality of the work before deciding to include it in the paper, by speaking directly with the researchers to understand the design, implementation and limitations of their studies. This is now discussed in the limitations.

*5. Are there particular reasons for not searching for grey literature?*

 As noted in the response to question 1, we did include grey literature from EMBASE and our expert network. This has now been clarified in the manuscript. We also acknowledge that our grey literature search may not have been comprehensive, and have added this as a limitation.  

*6. In studies with multiple antigens, what informed the choice of antigen that was included in the analysis?*

 This is a good question and something we discussed at length. To maintain focus in the manuscript, we examined which were the most commonly reported antigens and doses across the studies, and opted to use those for the main analyses. However, we also had research questions about whether recall varied depending on the antigen or dose in question – these questions could only be answered by papers reporting on multiple antigens and/or doses. For those analyses, we considered all antigens and doses presented in the study.

*7. The authors should provide the complete search strategy used for one of the two databases, preferably Medline.*

 The detailed search syntax is included in the supplemental materials on OSF.

 Thank you again for your review of our article.

# Peer review: Reviewer response for version 1

**DOI:** 10.21956/gatesopenres.14015.r27020

**Published:** 2019-04-09

**Authors:** Celina M. Hanson

**Affiliations:** 1 United Nations Children's Fund (UNICEF -United Nations International Children's Emergency Fund ), New York, NY, USA

This is a great systematic review by Dansereau and colleagues examining the agreement of vaccination recall data, home-based records, health facility records, BCG scars and serological data in LMICs and I approve this article. This review is an updated and expanded version from one published by Miles *et al. *in 2013 and focuses on LMICs with some additional analyses. This article is timely given WHO's 2017 recommendation listing the reliability of vaccination recall a high research priority and given that the previous Miles *et al.* article only elicited 5 LMICs.

 The article is written clearly and the methodology is also mostly clear and seems like it could be easily replicated. What was not entirely clear was the inclusion of grey literature as the article states the initial search for peer reviewed articles. However, manual reference searches and word-of-mouth experts provided means to include other articles; it was not entirely clear that grey literature was an inclusion criteria until reviewing the list of included articles. The article does list as a limitation that it may have missed some articles; however, one recommendation is the inclusion of specific antigen names in the search terms as this may have elicited more articles.

 Overall, this was a well-written and timely article with interesting and thorough analyses including the following variability factors: coverage level, antigen, number of doses, age of child, vaccine schedule complexity and demographic factors.

I confirm that I have read this submission and believe that I have an appropriate level of expertise to confirm that it is of an acceptable scientific standard.

# Response

**Published:** 2019-12-21

**Authors:** Emily Dansereau

Thank you very much for the time and thought given to review our article. 

 The reviewer is correct - we did include grey literature from EMBASE and our expert network, and this was not properly described in the manuscript. This has now been clarified in the manuscript. We also acknowledge that our grey literature search may not have been comprehensive, and have added this as a limitation.  

 Many thanks again for the thoughtful review.
